# Assessment of the Significance of LP-PLA2, DPYSL2, and 8-OHdG in the Oncological Diagnosis of Patients with Brain Tumors and Vitamin D Deficiency

**DOI:** 10.3390/ijms26167990

**Published:** 2025-08-19

**Authors:** Bartłomiej Gromadzki, Michał Wiciński, Zygmunt Siedlecki, Łukasz Rzepiński, Anna Fajkiel-Madajczyk

**Affiliations:** 1 Department of Pharmacology and Therapeutics, Collegium Medicum in Bydgoszcz, Nicolaus Copernicus University in Toruń, 85-090 Bydgoszcz, Poland; michal.wicinski@cm.umk.pl (M.W.); anna.fajkiel@cm.umk.pl (A.F.-M.); 2 Department of Neurosurgery, Neurotraumatology and Pediatric Neurosurgery, Collegium Medicum in Bydgoszcz, Nicolaus Copernicus University in Toruń, 85-094 Bydgoszcz, Poland; siedlecki@cm.umk.pl; 3 Department of Neurology, The 10th Military Research Hospital and Polyclinic, 85-681 Bydgoszcz, Poland; lukrzepinski@gmail.com

**Keywords:** brain tumors, vitamin D, Lp-PLA2, DPYSL2, 8-OHdG

## Abstract

Brain tumors are a significant medical problem. This study aimed to assess the potential significance of DPYSL2, 8-OHdG, and LP-PLA2 in the oncological diagnosis of patients with brain tumors who have a vitamin D deficiency. To this end, we measured the concentrations of LP-PLA2, DPYSL2, and 8-OHdG in 62 patients with different types of brain tumors who exhibited vitamin D deficiency. No significant variations were observed that would enable the differentiation of different types of intracranial tumors. However, we found a correlation between the concentrations of LP-PLA2 and DPYSL2 and tumor size (in the GBM and brain metastases groups). Additionally, we found a correlation between Lp-PLA2 and DPYSL2 levels in patients with GBM and brain metastases. In the control group, we identified correlations between 8-OHdG and Lp-PLA2, as well as between vitamin D levels and 8-OHdG. Further research is needed to fully comprehend the examined issues.

## 1. Introduction

Central nervous system (CNS) tumors represent a significant medical problem, and understanding the factors that influence tumor development is of paramount importance. CNS tumors have an incidence rate of 25.34 per 100,000 individuals. Among this group of tumors, malignant tumors have an incidence rate of 6.89 per 100,000, while benign tumors have an incidence rate of 18.46 per 100,000 [1]. Glioblastoma accounts for the highest percentage of malignant brain tumors. Glioblastoma (GBM) is a malignant, primary brain tumor classified as grade 4 according to the WHO classification. GBM is the most common primary malignant tumor of the central nervous system, constituting about 50% of all malignant CNS tumors. It is the most lethal among the malignant CNS tumors and has a poor prognosis, with a median survival of 9 months [1]. Brain metastases are tumors that develop outside the CNS and spread to the brain, mainly through the bloodstream (rarely by infiltration through continuity with surrounding anatomical structures). Brain metastases are the most common malignant brain tumors diagnosed in adults. Approximately 30% of adults with malignant tumors will develop brain metastases [2,3]. Meningiomas constitute a benign group of tumors arising from the meningothelial cells of the arachnoid mater. Meningiomas are the most common primary brain tumor in adults, occurring in approximately 1% of the population. They account for 56.8% of non-malignant CNS tumors [1].

Vitamin D deficiency has been described as a global problem [4]. However, precise epidemiological data on patients with brain tumors and vitamin D deficiency are not available. Vitamin D deficiency is characterized by serum levels below 20 ng/mL, while normal levels range from 40 to 60 ng/mL [5]. Vitamin D and its analogs have cancer-protective effects, modulating the metabolism of tumor suppressors and reducing cancer mortality [6,7]. Importantly, vitamin D has potential anti-tumor activity against GBM by promoting cell cycle arrest, inducing apoptosis, increasing vitamin D receptor-mediated autophagy, and exerting anti-migratory and anti-invasive effects [8]. Elmaci et al. reviewed epidemiological and cellular studies to determine the anticancer efficacy of vitamin D analogs against GBM and assessed their potential synergy with retinoic acid and temozolomide [9]. They found that winter delivery is associated with a higher risk of GBM. The neurosurgical procedure of GBM resection in winter increases mortality; this may be associated with reduced exposure to sunlight, which is necessary for the conversion of cholecalciferol to vitamin D. Comparative analyses of blood samples have shown that higher levels of calcidiol are associated with a lower risk of GBM in older men. According to Elmaci et al., vitamin D supplementation reduced mortality in patients with GBM compared with patients who did not take the supplement. Vitamin D receptor expression is associated with good GBM prognosis.

This study aimed to determine whether selected markers—lipoprotein-associated phospholipase A2 (Lp-PLA2), dihydropyrimidinase-like 2 (DPYSL2), and 8-hydroxy-2′-deoxyguanosine (8-OHdG)—can differentiate specific types of brain tumors in patients with vitamin D deficiency, i.e., whether these markers have diagnostic significance in oncology. The choice of these specific biomarkers is justified by their association with the pathomechanisms of the neoplastic process and by existing reports indicating their potential as biochemical markers in oncology. Further studies aim to evaluate their clinical utility and to identify the most promising markers for diagnostic purposes.

LpPLA2 plays a significant role in the development of neoplastic diseases through several pathways and pathophysiological mechanisms, including the release of arachidonic acid. The release of arachidonic acid by Lp-PLA2 leads to the production of reactive oxygen species through eicosanoid metabolism [10]. Reactive oxygen species can damage DNA, lead to genetic mutations, and alter the function of tumor suppressor genes (p53), which promote tumor development. Lp-PLA2 action also leads to changes to the structure of cell membranes, which can affect the functioning of receptors and ion channels. These changes can facilitate the proliferation of cancer cells [11]. LpPLA2 also plays a role in tumorigenesis by affecting the tumor microenvironment. The tumor microenvironment is a complex network of cells and molecules that supports the survival and growth of cancer cells. During a chronic inflammatory state in certain tumors, elevated levels of Lp-PLA2 lead to the release of additional pro-inflammatory mediators, creating a microenvironment conducive to tumor growth. Lp-PLA2 is involved in the recruitment of inflammatory cells to the tumor site. These cells can support tumor growth by secreting growth factors and pro-inflammatory cytokines [12]. Lp-PLA2 may influence the immune system, which in some cases may promote tumor development. In chronic inflammation, which promotes tumor development, Lp-PLA2 may modulate the activity of T cells and other immune cells [12]. Excessive inflammatory responses can inhibit the body’s ability to detect and eliminate cancer cells, weakening the immune response and promoting tumor growth. Lp-PLA2 may also participate in processes that weaken T lymphocyte function, reducing the body’s ability to defend itself against cancer cells [12,13]. Additionally, LpPLA2 plays an important role in angiogenesis, which is the formation of new blood vessels crucial for tumor growth [14]. The products of Lp-PLA2 activity, such as prostaglandins, stimulate angiogenesis, enabling tumors to develop further by providing access to oxygen and nutrients [10].

Reports have indicated the involvement of DPYSL2 in oncogenic processes; however, its role remains poorly understood. DPYSL2 promotes axonal growth, influences signaling processes within the CNS, and is correlated with poor GBM prognosis. DPYSL2 is overexpressed in glioblastoma stem cells, and its inhibition may have potential antitumor effects [15]. Furthermore, studies have demonstrated the involvement of DPYSL2 in the development of drug resistance and metastasis [16,17]. In breast cancer, a correlation has been demonstrated between mesenchymal markers and DPYSL2 expression. Additionally, deletion of the DPYSL2 gene in cancer cells resulted in a significant reduction in their migration and invasiveness, as well as tumor progression and metastasis [18].

8-OHdG is the product of oxidative damage to 2′-deoxyguanosine. It may serve as a useful marker of increased oxidative stress, a process associated with carcinogenesis. 8-OHdG levels are elevated in various cancer tissues and may be considered a predictor of the prognosis of most solid tumors, including those developing in the CNS [19].

## 2. Results

The 62 patients included in the study were divided into three experimental groups based on their diagnosis: GBM WHO IV, brain metastases, and meningioma. A control group was also included. Table 1 shows the number and sex distribution in each group.

The patients had no statistically significant differences in age. The mean ages of the patients by group were 62.67 ± 2.92 years for the GBM WHO IV group, 63.55 ± 2.1 years for the brain metastases group, 65.13 ± 3.56 years for the meningioma group, and 67.11 ± 3.1 years for the control group.

**Figure 1:**
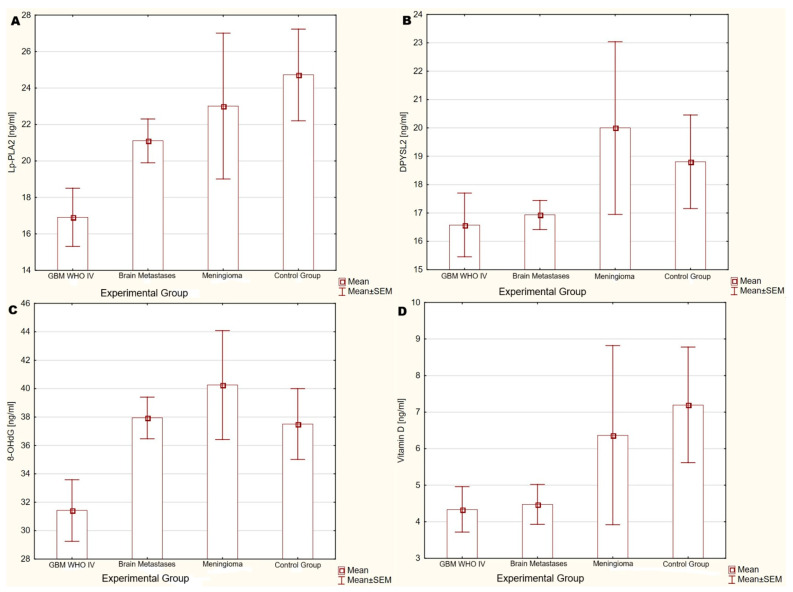
Mean ± SEM serum concentrations of (**A**) Lp-PLA2, (**B**) DPYSL2, (**C**) 8-OHdG, and (**D**) vitamin D in each group (*p* > 0.05 for all comparisons).

Mean ± SEM values for Lp-PLA2, DPYSL2, 8-OHdG, and vitamin D concentrations were calculated for each group. Due to the lack of normal distribution, the groups were compared using the Kruskal–Wallis test, with post hoc analysis and multiple testing correction using the Benjamini–Hochberg procedure. There were no statistically significant differences (*p* > 0.05) between the groups in terms of Lp-PLA2, DPYSL2, 8-OHdG, and vitamin D concentrations. Figure 1 illustrates the concentrations of Lp-PLA2, DPYSL2, 8-OHdG, and vitamin D in each group.

The study also assessed whether there was a correlation between Lp-PLA2, DPYSL2, 8-OHdG, and vitamin D concentrations and sex or age. The analyses revealed a statistically significant moderate positive correlation between sex and Lp-PLA2 concentration (R = 0.46; *p* = 0.04) in patients with brain metastases and a strong negative correlation between DPYSL2 and sex (R = −0.85; *p* = 0.008) in patients with meningioma. In contrast, the control group exhibited a statistically significant moderate negative correlation between age and vitamin D concentration (R = −0.53; *p* = 0.02).

**Figure 2:**
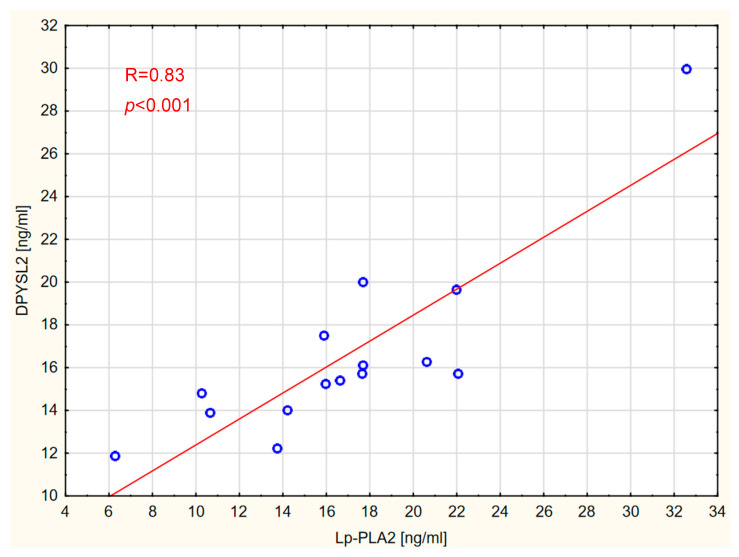
Correlation between Lp-PLA2 and DPYSL2 levels in the GBM WHO IV group.

**Figure 3:**
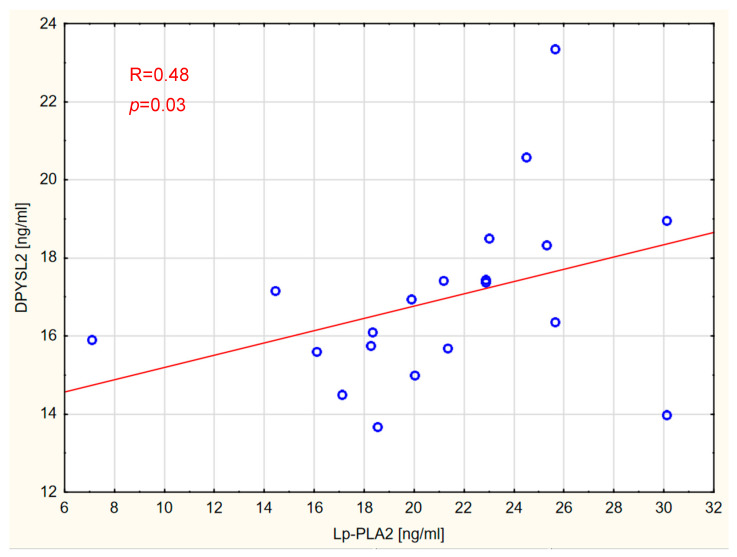
Correlation between Lp-PLA2 and DPYSL2 levels in the brain metastases group.

The presence of correlations between the concentrations of individual markers and between the measured markers and vitamin D was also assessed in all groups. The analyses revealed correlations between the concentration of Lp-PLA2 and DPYSL2 in patients with GBM (Figure 2) and brain metastases (Figure 3).

**Figure 4:**
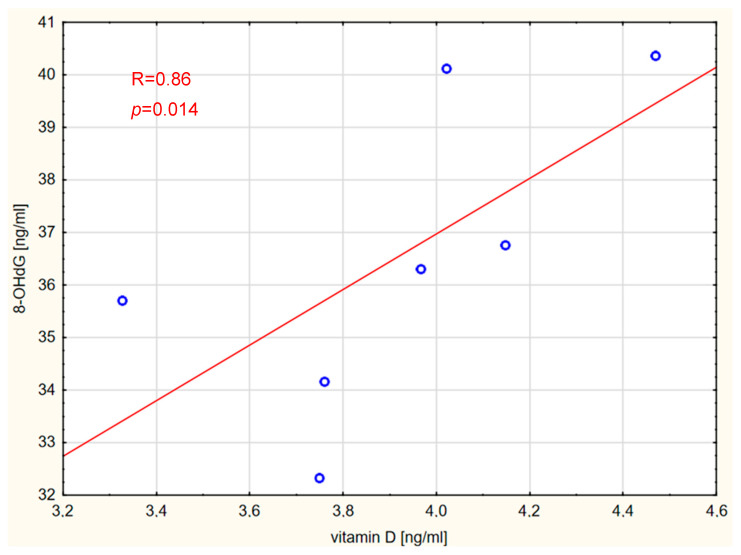
Correlation between 8-OHdG and vitamin D levels in the meningioma group.

A strong positive correlation was found between the concentration of 8-OHdG and vitamin D in patients with meningioma (Figure 4).

**Figure 5:**
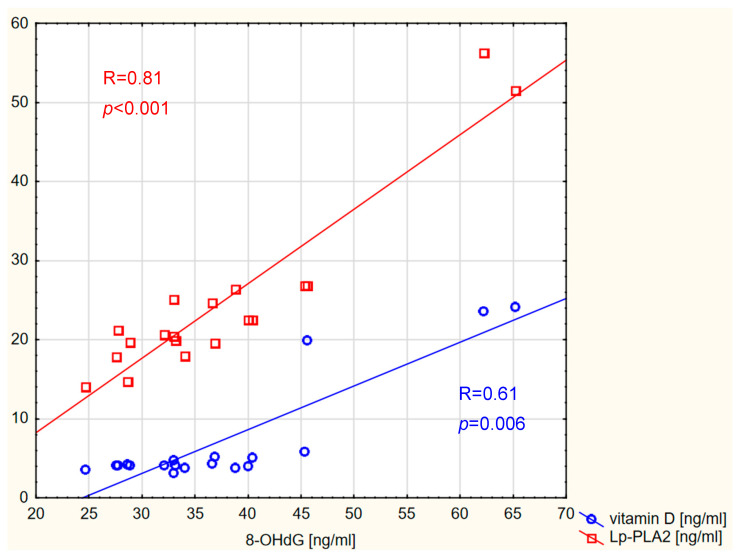
Correlation between 8-OHdG and vitamin D and Lp-PLA2 levels in the control group.

Correlations were also detected between 8-OHdG and vitamin D concentrations, as well as between 8-OHdG and Lp-PLA2 concentrations, in the control group, as shown in Figure 5.

**Figure 6:**
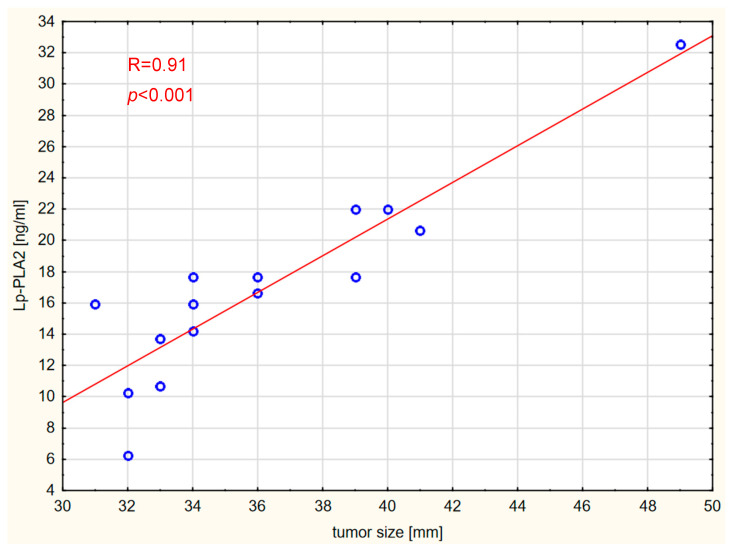
Correlation between Lp-PLA2 levels and tumor size in the GBM WHO IV group.

**Figure 7:**
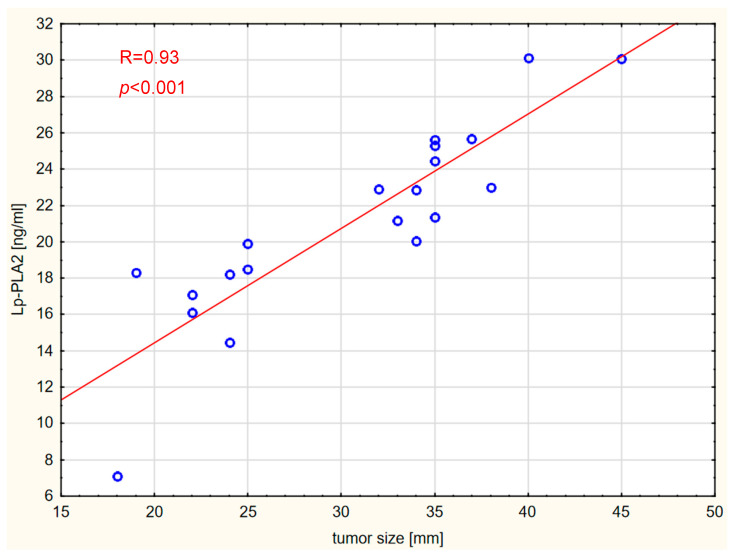
Correlation between Lp-PLA2 levels and tumor size in the brain metastases group.

**Figure 8:**
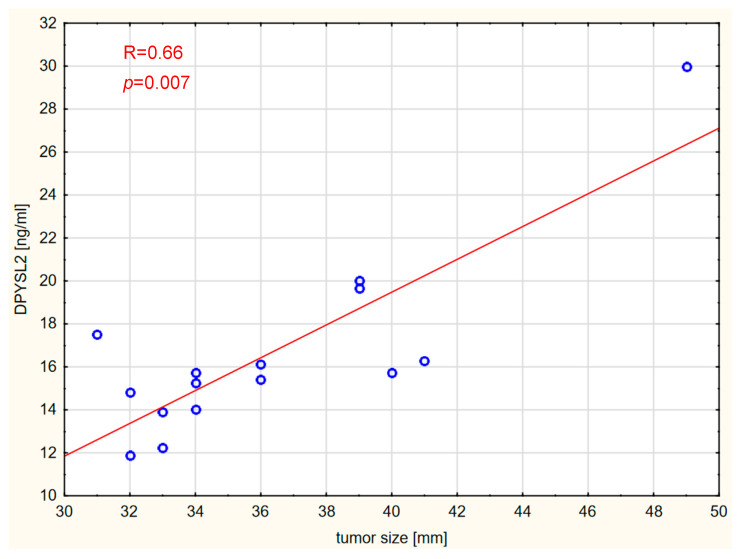
Correlation between DPYSL2 levels and tumor size in the GBM WHO IV group.

**Figure 9:**
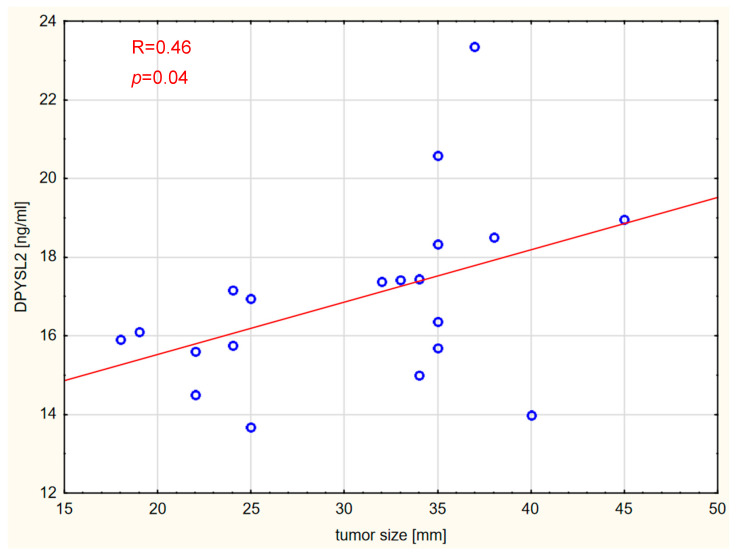
Correlation between DPYSL2 levels and tumor size in the brain metastases group.

Correlations between the markers and tumor size, expressed in millimeters, were also assessed. Vitamin D concentration was not significantly correlated with tumor size in any of the analyzed groups. Interestingly, tumor size was strongly positively correlated with Lp-PLA2 and DPYSL2 concentrations in the GBM and brain metastases groups. The results are shown graphically in Figure 6, Figure 7, Figure 8 and Figure 9.

**Figure 10:**
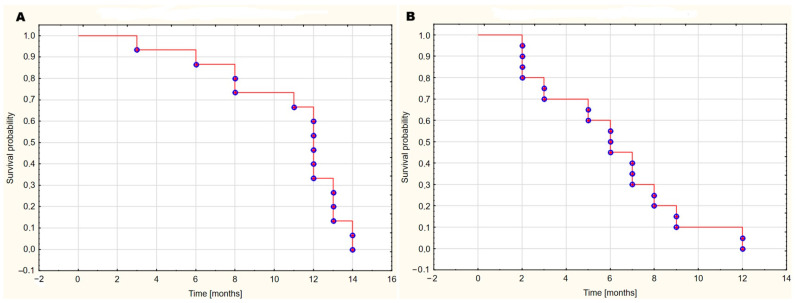
Kaplan–Meier survival analysis for the GBM WHO IV (**A**) and brain metastases (**B**) groups.

The median survival time in the GBM WHO IV group was 12 months, meaning that half of the patients survived at least 12 months after surgery. The minimum survival time in this group was 3 months, and the maximum was 14 months. In the brain metastases group, the median survival time was 6 months, with a minimum survival time of 2 months and a maximum of 12 months. Figure 10 illustrates the Kaplan–Meier survival analysis for these groups.

## 3. Discussion

Our study aimed to assess whether serum concentrations of LpPLA2, DPYSL, and 8-OHdG in patients with vitamin D deficiency can differentiate specific types of brain tumors and if they have potential diagnostic significance. This is the first study conducted on such a patient group, specifically, patients with brain tumors and vitamin D deficiency. Measuring vitamin D levels in cancer patients aimed to elucidate its role in the disease’s pathogenesis. The widespread deficiency of vitamin D in individuals with cancer makes this area of research particularly interesting [20]. Vitamin D has strong anti-inflammatory properties that may influence the activity of enzymes associated with inflammation. Studies have shown that vitamin D reduces the production of pro-inflammatory cytokines such as interleukin-6 (IL-6) and tumor necrosis factor-alpha (TNF-α) [21,22]. It has also been established that vitamin D deficiency enhances inflammation (increase in C-reactive protein (CRP)) [23]. Chronic inflammation, in turn, is a key factor in tumorigenesis and metastasis [24].

The study included three groups of patients—each differing in tumor type and origin—and three markers. The first group consisted of patients with primary (originating from brain tissue) malignant brain tumors, namely, glioblastoma WHO IV. The second group included patients with secondary brain tumors, i.e., metastases. The third group included patients with meningioma. Meningioma differs from the other tumors in several ways. Firstly, it is a benign tumor. Secondly, unlike the other tumors, it does not develop in the brain parenchyma (intra-axial) but rather from arachnoid cells (extra-axial change) and causes brain compression. The final group consisted of control participants. Concentrations of the markers in the serum were evaluated to determine whether statistically significant differences existed between the groups and whether these differences could be used to differentiate the groups. In summary, no significant variations were observed that would enable differentiation between the groups. However, several interesting correlations were identified. Lp-PLA2 levels were strongly correlated with tumor size in patients with GBM and brain metastases. DPYSL2 concentrations were also correlated with tumor size in both of these patient groups. In contrast, no correlation was found between tumor size and 8-OHdG levels. A positive correlation was observed between Lp-PLA2 and DPYSL2 levels in patients with GBM and brain metastases. In patients with meningioma, a positive correlation was detected between 8-OHdG and vitamin D concentrations. Furthermore, correlations between 8-OHdG and Lp-PLA2 levels, as well as between vitamin D and 8-OHdG levels, were identified in the control group. A statistically significant moderate positive correlation was found between sex and Lp-PLA2 concentration in the group of patients with brain metastases, while a strong negative correlation was observed between DPYSL2 levels and sex among patients with meningioma. Conversely, a negative correlation was identified between age and serum vitamin D levels in the control group.

Lp-PLA2 is a calcium-independent phospholipase A2 (PLA2) enzyme secreted mainly by macrophages or activated platelets and classified as group VIIA PLA2 (PLA2G7) [25]. Lp-PLA catalyzes the hydrolysis of cellular glycerophospholipids at the sn-2 position, leading to the release of oxidized non-esterified fatty acids and arachidonic acid (AA) [26]. The metabolism of free AA generates prostanoids (PGD2, PGE2, PGF2α, PGI2) and thromboxane A2 that are involved in cellular proliferation, immune suppression, cancer-associated inflammation, tumor progression, and metastasis [27,28]. Lp-PLA2 is a well-recognized biomarker of oxidative stress, inflammation, and atherosclerosis that is distributed in both intracellular and extracellular fluids [29]. Interestingly, the PLA2G7 gene, which encodes Lp-PLA2, plays a crucial role in the oncogenesis of various human cancers, including those within the CNS [30]. Furthermore, increased PLA2 activity was observed in glioblastoma tissue, promoting proliferation and viability of tumor cells [31]. Studies on colorectal cancer have suggested that high PLA2G7 enzyme activity plays a key role in the development of the disease. Experiments on mice carrying the ApcMin/+ mutation showed that the removal of this enzyme reduces the number of intestinal polyps and limits tumor formation [32]. Elevated PLA2G7 levels have been found in patients with cachexia as well as colorectal and pancreatic cancer [33].

PLA2G7 plays a regulatory role in the Wnt signaling pathway in breast cancer [34]. Furthermore, macrophages with high levels of PLA2G7 in hepatocellular carcinomas have a strong immunosuppressive effect, inhibiting the activation of CD8+ T lymphocytes, indicating a key role of PLA2G7 in modulating the immune response around the tumor [35]. Importantly, our study revealed that tumor size was strongly positively correlated with serum Lp-PLA2 concentrations in patients with gliomas and metastatic CNS tumors. These findings may reinforce the potential significance of Lp-PLA2 as a serum biomarker of progression in the dimensions of malignant brain tumors.

Reports on the role of DPYSL2 in brain tumors are relatively scarce. The expression level of DPYSL2 varies depending on the tumor type [36,37,38]. Zottel et al. [39] demonstrated that DPYSL2 expression levels were significantly higher in GBM cells than in astrocytes. The authors suggested that this marker could be used to differentiate GBM from healthy brain tissue. Furthermore, it has been shown that DPYSL2 plays a role in GBM oncogenesis by participating in the suppression of HOXA11 [15]. Higher concentrations of DPYSL2 have been reported in tumor tissues compared with surrounding tissue, as well as in colorectal carcinoma [38]. In contrast, lower DPYSL2 expression has been observed in lung adenocarcinoma (LUAD) tissues [36]. Low levels of DPYSL2 have also been associated with poorer prognosis. Lingling Zu et al. [37] found that downregulation of DPYSL2 expression is linked to increased distant metastasis in non-small cell lung cancer (NSCLC). In our study, there was no significant difference in DPYSL2 serum concentrations between the patient and control groups. However, we observed a correlation between serum DPYSL2 levels and tumor size (both primary and secondary brain tumors), but not meningiomas. Similarly to Lp-PLA2, this may suggest a potential role as a marker for tumor progression associated with tumor size. Additionally, the study results demonstrated a correlation between serum levels of Lp-PLA2 and DPYSL2 in patients with GBM, brain metastases, and in the control group. Again, such a correlation was not observed in patients with meningioma. We did not identify studies assessing the potential correlation between Lp-PLA2 and DPYSL2. It has been demonstrated that DPYSL2 may influence oncogenesis by activating the JAK1/STAT3 pathway [18]; these researchers suggested that the C-terminal domain of DPYSL2 interacts with JAK1, leading to the phosphorylation of STAT3 and its translocation into the nucleus, which enables the regulation of specific target genes. Evidence indicates that excessive activation of STAT3 plays a broad role in the development of many cancer types, including brain tumors, lung cancer, breast cancer, melanoma, and metastasis formation [40]. The JAK/STAT signaling pathway can be activated by various pro-inflammatory factors, including IL-6, IL-11, and IL-13 [41]. Lp-PLA2 influences the levels of pro-inflammatory cytokines through its role in promoting inflammatory processes [42], which may potentially impact the JAK/STAT pathway. Furthermore, Yao et al. [43] observed that silencing Lp-PLA2 results in the deactivation of the JAK2/STAT3 signaling pathway. Peng et al. also found that PLA2G7 is involved in regulating the JAK/STAT pathway in bladder cancer, and silencing PLA2G7 expression results in significantly lower levels of p-STAT1, p-STAT2, and IRF1 proteins [44].

8-OHdG is a biomarker of oxidative stress. Large amounts of reactive oxygen species (ROS) and reactive nitrogen species (RNS) are secreted in cells experiencing chronic inflammation. If the quantity of these ROS/RNS is substantial, it may overwhelm the antioxidant mechanisms, leading to oxidative damage to nucleic acids, lipids, and proteins [45]. Consequently, this process may result in genetic alterations and the initiation of oncogenesis. However, elevated levels of ROS may play the opposite role—they can lead to tumor suppression by inhibiting proliferation and inducing cell death [46]. Elevated concentrations of 8-OHdG have been observed in various tumor types, including gastric, colorectal, and ovarian cancers [45]. Tuzgen et al. [47] demonstrated significantly higher levels of 8-OHdG in glioblastoma versus control tissues. A meta-analysis [19] found that 8-OhdG was overexpressed in various tumor types, and high 8-OhdG expression was a marker of poor prognosis; an exception was observed in a few tumors, including breast cancer. No significant differences in serum 8-OHdG levels were detected between the different groups in our study.

Our study was conducted on patients with vitamin D deficiency. We observed a correlation between vitamin D and 8-OHdG levels in the control group and subjects with meningioma. Additionally, a negative correlation was found between age and serum vitamin D levels in the control group. No significant association was observed between vitamin D levels and the studied markers in patients with malignant brain tumors (primary or secondary). The identified correlation between vitamin D and 8-OHdG levels differs from reports in the literature. Low vitamin D levels have been associated with high levels of 8-OHdG [48]. A reduction in 8-OHdG levels following vitamin D supplementation was observed in patients with colorectal adenoma [49].

This study has several limitations. One of the main limitations is the small sample size. The results may be affected by other comorbidities and the medications used during treatment. Additionally, the heterogeneity among the patients with brain metastases—due to the diversity of histopathological diagnoses—introduces variability and complicates the interpretation of the results. Furthermore, marker measurements were performed preoperatively, which does not account for potential changes in their levels over time. Another limitation of our study is that the control group included patients with spinal disk disease and vitamin D deficiency, which may have affected the results, since these patients also experience chronic inflammation, unlike healthy individuals. This study does not provide all the answers, and further research is needed to fully comprehend the examined issues.

## 4. Materials and Methods

### 4.1. Bioethics

The study was conducted in accordance with the Declaration of Helsinki and approved by the Bioethics Committee of Collegium Medicum in Bydgoszcz, Nicolaus Copernicus University in Toruń, Poland (approval number KB 115/2021; approval date 16 February 2021). Written informed consent was obtained from all participants involved in the study.

### 4.2. Study Cohort

Sixty-two patients from the Department of Neurosurgery at the Specialist City Hospital in Toruń were included in the study. The experimental group, consisting of 43 subjects, included patients who qualified for surgical treatment for brain cancer. The subjects were divided into three subgroups based on their diagnosis: GBM WHO IV, brain metastases, or meningioma. The control group, which included 19 subjects, consisted of non-cancer patients and non-smokers who were not treated for metabolic diseases and were undergoing treatment in the Department of Neurosurgery for spinal disk disease. The study was conducted between September 2021 and February 2022 at the Department of Neurosurgery of the Specialist City Hospital in Toruń, where blood samples were collected from patients for further analysis.

### 4.3. Measurements

A venous blood sample was collected from each patient. The material for the study was prepared following a standard laboratory procedure, including preparation of the serum, freezing the material at −20 °C, and transporting it on dry ice to the Department of Pharmacology and Therapeutics, Collegium Medicum in Bydgoszcz, where it was stored at −70 °C until analysis. The tested parameters—Lp-PLA2, DPSYL2, 8-OHdG, and vitamin D—were analyzed using immunoenzymatic assays (ELISA) according to the manufacturer’s instructions (Sunredbio (SRB) Technology, Shanghai, China). All studies used an EPOCH microplate spectrophotometer (BioTech, Santa Clara, CA, USA).

### 4.4. Statistical Analysis

Data are presented as mean ± standard error of the mean (SEM). The normality of distribution was checked using the Shapiro–Wilk test. Due to the distribution of the variables, the levels of analyzed markers between groups were compared using the Kruskal–Wallis test with post hoc analysis. Multiple testing correction was performed using the Benjamini–Hochberg procedure. Spearman’s rank correlation was used to assess the correlation between different parameters. *p* < 0.05 indicates statistically significant differences. Survival analysis for the GBM WHO IV and brain metastases groups was conducted using the Kaplan–Meier method. All statistical analyses were conducted using STATISTICA ver. 13.3 (TIBCO Software Inc., Palo Alto, CA, USA).

## Figures and Tables

**Table 1:** Number and sex distribution in each group.

Group				
	Experimental Group			Control Group
	GBM WHO IV	Brain Metastases	Meningioma	Spinal Disk Disease
*n*	15	20	8	19
Females	4	9	5	12
Males	11	11	3	7

## Data Availability

The original contributions presented in this study are included in the article. Further inquiries can be directed to the corresponding author.

## Abbreviations

8-OHdG	8-hydroxy-2′-deoxyguanosine
AA	arachidonic acid
CNS	central nervous system
CRP	C-reactive protein
DPYSL2	dihydropyrimidinase-like 2
GBM	glioblastoma multiforme WHO 4
IL-6	interleukin-6
IL-11	interleukin-11
IL-13	interleukin-13
JAK1/STAT3	Janus kinase 1/signal transducer and activator of transcription 3
Lp-PLA2	lipoprotein-associated phospholipase A2
LUAD	lung adenocarcinoma
NSCLC	non-small cell lung cancer
PGE2	prostaglandin E2
PGD2	prostaglandin D2
PGI	prostaglandin I
PLA2	phospholipase A2
PLA2G7	group VIIA phospholipase A2
RNS	reactive nitrogen species
ROS	reactive oxygen species
TNF-α	tumor necrosis factor-alpha
